# Ingestion of microplastics and microfibers by the invasive blue crab *Callinectes sapidus* (Rathbun 1896) in the Balearic Islands, Spain

**DOI:** 10.1007/s11356-023-30333-x

**Published:** 2023-11-04

**Authors:** Montserrat Compa, Esperança Perelló, Antoni Box, Victor Colomar, Samuel Pinya, Antoni Sureda

**Affiliations:** 1 Research Group in Community Nutrition and Oxidative Stress, University of Balearic Islands, 07122 Palma, Balearic Islands Spain; 2 Interdisciplinary Ecology Group, University of the Balearic Islands, Ctra. Valldemossa Km 7,5, 07122 Palma, Balearic Islands Spain; 3 Department of Agricultura, Ramaderia, Pesca, Caça I Cooperació Municipal, Consell Insular d’Eivissa, 07800 Eivissa, Spain; 4 Consortium for the Recovery of Fauna of the Balearic Islands (COFIB), Government of the Balearic Islands, Ctra. Palma-Sineu Km 15.4, 07141 Santa Eugènia, Balearic Islands Spain; 5 Health Research Institute of Balearic Islands (IdISBa), 07120 Palma, Spain; 6 CIBER Fisiopatología de La Obesidad y Nutrición (CIBEROBN), Instituto de Salud Carlos III (ISCIII), 28029 Madrid, Spain

**Keywords:** Microplastics, *Callinectes sapidus*, Invasive species, Monitoring

## Abstract

The blue crab *Callinectes sapidus* Rathbun, 1896 is native to the western coasts of the Atlantic Ocean and is currently considered an invasive species in the Mediterranean Sea. In this study, we examined the stomach contents of *C. sapidus* to determine the frequency of occurrence of microplastics (MPs) and microfibers (MFs) in the Balearic Islands archipelago in the western Mediterranean Sea. A total of 120 individuals were collected from six locations between 2017 and 2020. Overall, 65.8% of the individuals had MPs and/or MFs particles with an average of 1.4 ± 1.6 particles ind.^−1^ of which an average of 1.0 ± 1.3 items ind.^−1^ were MFs and an average of 0.4 ± 0.8 items ind.^−1^ were MPs. In terms of type, fragments were the dominant type of MPs and the most common size of items ranged from between 0.5 and 1 mm (40%) followed by 1–5 mm (31%). The most prevalent polymers were low-density polyethylene (39%) and high-density polyethylene (26%). In terms of links to human activities, MP ingestion was positively correlated with an increase in drain pipes, whereas MF ingestion was positively correlated with an increase in sewage pipelines, providing evidence of potential sources and the bioavailability of these particles in various environments. This study confirms the widespread presence of MP and MF particles, even in areas that are currently managed under different protection statuses, in the stomach contents of invasive blue crab species throughout coastal communities.

**Supplementary Information:**

The online version contains supplementary material available at 10.1007/s11356-023-30333-x.

## Introduction

In marine and terrestrial environments, especially those close to the shoreline, microplastics (MPs, < 5 mm) and microfibres (MFs, < 5 mm) of synthetic origin represent an omnipresent hazard (Compa et al. 2020; Rios-Fuster et al. 2022, 2019; Sanchez-Vidal et al. 2021). An increasing number of coastal ecosystems are identified as possible sinks for MPs and MFs, including estuaries, salt marshes and wetlands (Lloret et al. 2021). MPs have been reported to be preserved in marine sediments, especially in coastal areas, and their presence has the potential to have a severe impact on species, particularly benthic fauna, which are essential to bottom-up processes including energy transfer and nutrient remineralisation (Mason et al. 2022). In particular, decapods, specifically crabs that live in these regions, are exposed to both abiotic and biotic compartments, making them an ideal candidate for research on the role they play in MP retention and toxicity. In terms of diet, the ingestion of these items was variable in omnivorous crabs (0 to 117 items individual^−1^) (Piarulli et al. 2019) while in a more predatory crab, less variation was observed with all individuals ingesting between 43 and 50 items individual^−1^ (Aliko et al. 2022). Moreover, under experimental studies, chronic acute exposure to MPs has been observed to harm filter-feeding crabs (Urbina et al. 2023). The presence of these particles has been observed not only within coastal crab habitats in recent years all over the world, but also in various tissues such as the digestive system and gills (Zhang et al. 2021). Moreover, many species found to interact with MPs and MFs, are commercially important species (Ogunola et al. 2022). In addition to their interaction with these contaminants, crabs have been observed to alter ecosystems, such as a reduced cover of seaweed and algae from intensive grazing, resulting in more corals and fishes on reefs (Spadaro et al. 2021). Considering the prevalence of these particles in global habitats, it is important to evaluate this at a local scale.

In terms of MPs in the Balearic Islands, located in the western Mediterranean Sea, it has been noted that high abundances of plastics are present in coastal areas and are floating on the sea surface (Compa et al. 2020; Fagiano et al. 2022b) and accumulating in seafloor sediments (Alomar et al. 2016; Lombardo et al. 2022) with high abundances highlighted in coastal regions. Moreover, its occurrence has been observed in coastal fauna, such as the recreationally relevant fish species *Xyrichtys novacula* (Cohen-Sánchez et al. 2022) and the demersal catshark *Galeus melastomus* (Alomar and Deudero 2017) in addition to an overlap observed between marine debris on the seafloor and demersal species throughout the Balearic Islands shelf (Alomar et al. 2020). Benthic organisms have also been recognized as vulnerable species to MP ingestion, including sea urchins and sea cucumbers (Compa et al. 2022; Lombardo et al. 2022). In laboratory studies, evidence of the toxicity of crabs exposed to MPs over time included lower food intake, and growth energy (Watts et al. 2015) as well as a decrease in the energy budget of the crabs because of exposure (Urbina et al. 2023) has been observed. The prevalence of these items has not only been detected in occurring within organisms, but it has also been found to be stress-inducing, in addition to the incidence of persistent organic pollutants and heavy metals (Rochman et al. 2014; Salvaggio et al. 2019). For example, an increase in glutathione S-transferase (GST) activity was detected in *Mullus surmuletus*, which could suggest induction of detoxification systems (Alomar et al. 2017) while an increase in malondialdehyde (MDA) levels was observed in liver and brain tissue of the commercially important fish species *Engraulis encrasicolus*, and an increase in catalase (CAT) activity was detected in brain tissue of *M. surmuletus* and *Boops boops* (Capó et al. 2022). Furthermore, in fish chemical analyses revealed in the *Lepidopus caudatus*, there is presence of high content of components of plastics such as phthalates, and in particularly high quantities of diisodecyl phthalate, di(2-ethylhexyl) phthalate, bis-benzy lester phthalate, bis-butyl ester phthalate, and mono-*N*-butyl ester phthalate in different organs (Salvaggio et al. 2019). The prevalence of these items has been found to be stress-inducing, with increased levels of phthalates in marine organisms. There is a need for on-going monitoring in coastal areas, especially crab species because these chemicals can serve as indicators of ecosystem contamination, bioaccumulate through food webs, and potentially pose health risks to both crabs and higher trophic level consumers, including humans.

Native to the western Atlantic Ocean coasts, the blue crab, *Callinectes sapidus* Rathbun, 1896, is a significant fisheries resource in North and Central America; however, in the Mediterranean Sea, it is regarded as an invasive species. It was first reported in the Mediterranean Sea during the first half of the twentieth century, moving westward across the Israeli and Egyptian coastlines and the Gulf of Thessaloniki (Garcia et al. 2018) and continuing with its westward expansion along the coasts of France (Labrune et al. 2019), Spain (Box et al. 2020) and other regions of southern Europe and African coastlines (Mancinelli et al. 2017; Nehring 2011). In the Balearic Islands, two larvae of *C. sapidus* were first observed in July 2005 and October 2011, highlighting the influence of connectivity from currents within the Mediterranean Sea (Png-Gonzalez et al. 2021). Adult individuals of *C. sapidus* were first reported on 22 June 2017 on the islands of Mallorca and Menorca (Garcia et al. 2018) and have since expanded to salt ponds from an industrial salt production site and other coastal areas in Ibiza and Formentera (Box et al. 2020), thus confirming its extension throughout the archipelago. In the Mediterranean Sea, it is currently ranked among the top 100 worst alien invasive species (Streftaris and Zenetos 2006). In addition, to having good swimming abilities and being linked to high-fertility rates, its biological features make it a highly competitive species (Garcia et al. 2018). *C. sapidus* is known to be a highly mobile species, enhancing its ability to avoid potential predators in addition to reports of negative interactions with native fisheries resources, many of which are commercially significant (Mancinelli et al. 2017). Considering this, *C. sapidus* is an invasive species of concern that could have a significant harmful ecological impact on the surrounding environment.

In this study, we aimed to examine the stomach contents of the invasive crab species *C. sapidus* to (i) determine the frequency of occurrence of ingestion of MPs and MFs, (ii) identify the spatial distribution of ingestion and (iii) determine biological traits and human factors that can contribute to the prevalence of ingestion in the Balearic Islands.

## Materials and methods

### Study area

**Fig. 1:**
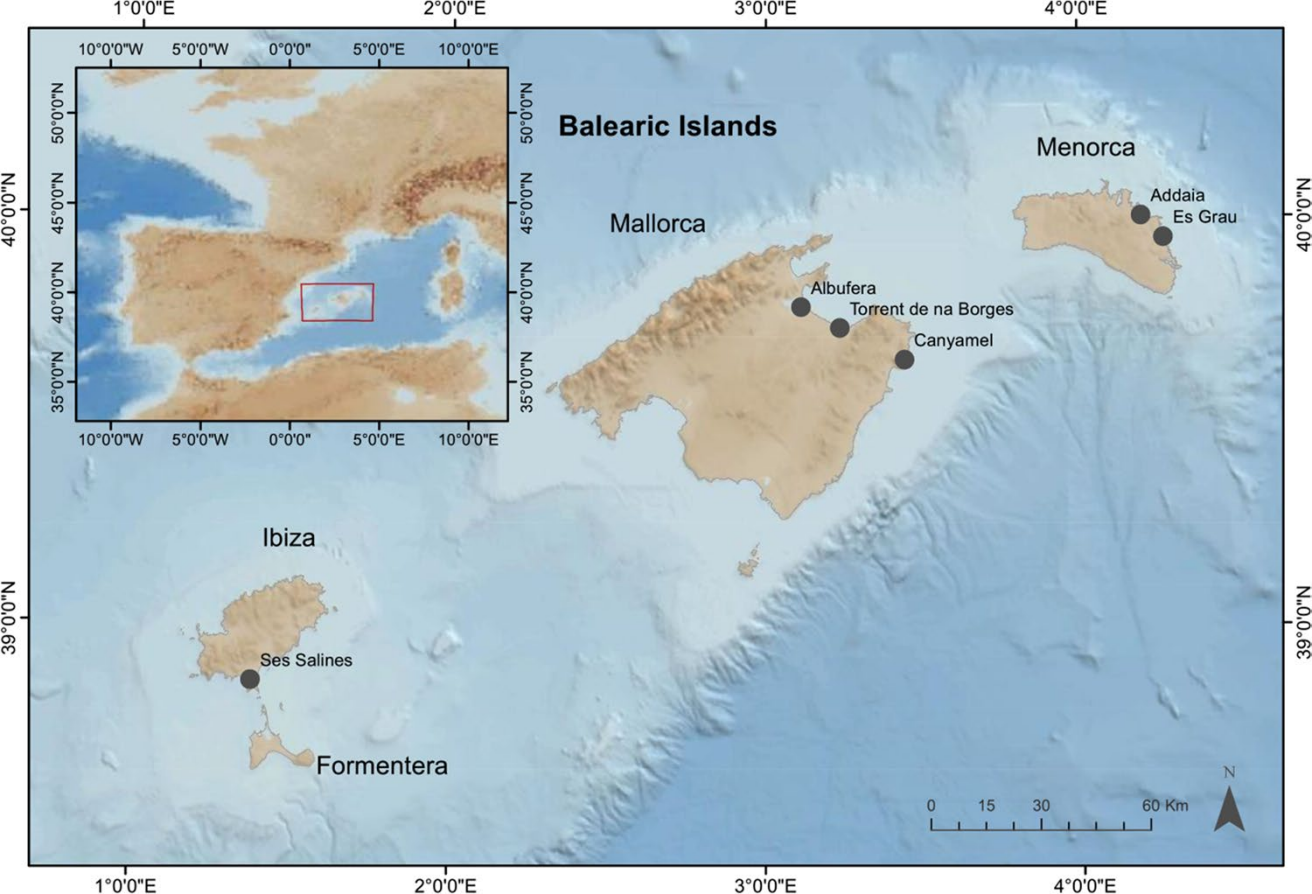
Map of the study area in the Balearic Islands and the six sampling locations for *C. sapidus* in the Balearic Islands. The inset map indicates the location of the Balearic Islands within the red box

The Balearic Island archipelago is an island chain consisting of four main islands located in the western Mediterranean Sea (Fig. 1). Individuals of *C. sapidus* were collected on the islands of Mallorca, Menorca and Ibiza from 2017 to 2020 using different capture methods including traps and nets in six locations.

In Mallorca, three locations were selected. The first two sites are located in the northern Bay of Alcúdia, the first within the Natural Park of S’Albufera, which is the largest wetland area in the Balearic Islands and is part of the EU Natura 2000 Network as a Special Area of Conservation (S’Albufera de Mallorca — ES5310125) within the Habitats Directive and a Special Protection Area within the Birds Directive. The second site is Torrent de na Borges, which is located on the eastern shores within the Bay of Alcúdia. It is the largest ephemeral stream in Mallorca and also forms part of the EU Natura 2000 Network as a Special Area of Conservation (Na Borges — ES5310029). The third site in Mallorca is Canyamel, which is a coastal city heavily influenced during the summer season by tourism. In Menorca, one site is located within the Natural Park of S’Albufera des Grau, which is a marshland and Special Protection Area and Special Area of Conservation protected under both Birds and Habitats Directives (S’Albufera des Grau — ES0000234) that is part of the Red Natura 2000 and is the centre of the Biosphere Reserve of Menorca. The second site in Addaia is also located within the Natural Park of S’Albufera des Grau; however, it is the third largest port area on the island. Finally, the Ses Salinas sampling site in Ibiza is a lagoon and a biodiversity hotspot with over 210 species known to forage. Ses Salines d’Eivissa i Formentera Natural Park is also part of Natura 2000 Network as a Special Protection Area and Special Area of Conservation protected under both Birds and Habitats Directives (Ses Salines d’Eivissa i Formentera — ES0000084). These six areas show the diversity of habitats in the Balearic Islands that originated in areas of special importance for endemic species in areas with potentially high anthropogenic influences, particularly during the summer months (July–September), taking into account the various protection statuses (Table 1).

**Table 1:** Summary of the sampling locations and a description of the physical characteristics and protection status of the sampling locations

Island	Sampling site	Physical characteristics	Habitats	Protection status
Mallorca	S’Albufera	Wetland	Grasslands and marshlands	Special Area of Conservation, RAMSAR wetland, Natural Park
	Canyamel	Torrent	Torrent mouth	-
	Torrent de na Borges	Lagoon	Wet meadows, dunes	Special Area of Conservation
Menorca	Addaia	Small Port Area	-	Biosphere Reserve; Special Area of Conservation; Special Protection Area of Birds; Natural Park
	Es Grau	Lagoon	Marshlands, wild olive woods, temporary ponds, dune systems, sea phanerogam prairies, coastal islets, etc	Biosphere Reserve; Natural Park; Special Area of Conservation; Special Protection Area of Birds
Ibiza	Ses Salines	Lagoon	Embryonic shifting dunes, *Posidonia oceanica* meadows, ponds, scrub, etc	Natural Park; Special Area of Conservation; Special Protection Area of Birds, RAMSAR wetland

### Sample dissection and digestion

At each location, 10 adult individuals for male and female were collected, and the following biometrics were recorded in situ: carapace length (cm), carapace width (cm), chela length (cm), and body weight (g). For each individual, the stomach was removed and stored in 96% alcohol and kept at 20 ºC. Once in the laboratory, the alcohol supernatant of all samples was first filtered through glass microfiber filters with a 1.2-μm pore size of 1.2 m and a diameter of 47 mm using a vacuum pump. The wet weight (g) for the stomach contents was weighed. Next, to remove organic material, the samples were chemically digested with 10% potassium hydroxide (KOH) in a proportion of 20 ml of KOH per gram of tissue at 60 °C for 24 to 48 h (Lombardo et al. 2022). Next, each sample was filtered through glass microfiber filters and left to dry at room temperature for at least 24 h before observation with a stereomicroscope for microplastic identification (Rios-Fuster et al. 2021).

### Microplastic analysis

The morphological and physical characteristics of each item were visually identified using a Leica stereomicroscope. Each filter was observed, and all visibly identifiable MP and MF items were classified by shape: fibre, fragment, pellet and colour: black, blue, white, transparent, red and other (blue-transparent, green, grey, orange and yellow). The length of the items was measured using ImageJ v0.5.7 (https://imagej.net/). For the polymer characterization, considering the size of all particles (APs), the identified items were characterised by μ-Attenuated Total Reflectance-Fourier Transform Infrared (μ-ATR-FTIR) (Bruker OPTICS, Ettlingen, Germany) to determine their polymer classification. Measurements were carried out using a wave number range between 400 and 4000 cm^−1^, 16 coadded scans and a spectral resolution of 4 cm^−1^, and the generated spectra were subjected to baseline correction to reduce noise and improve spectrum quality (Solomando et al. 2022). Then, all spectra were evaluated against both custom and JPI Oceans project BASEMAN (Primpke et al. 2019; Suaria et al. 2020) spectral databases. Similarities that exceeded a hit quality index (HQI) of 70% were regarded as acceptable. The University of the Balearic Islands’ Scientific/Technical Services assisted with the analysis.

### Data analysis

Microplastic data was expressed and analysed as the number of items per individual (items ind.^−1^) and the number of items per gram of wet weight (items g WW^−1^) of the stomach contents (Ogunola et al. 2022). In this study, microfibres (MFs) as those resembling textile material and microplastics (MPs) as all other types (fragments, filaments and granules) while the combination of both, for the purpose of this study, are all particles (APs). The first step was to identify differences between MFs and MPs using a non-parametric Mann–Whitney *U* test (MW). Next, the differences between locations for each by applying a Kruskal–Wallis test (KW) and the differences between sites were further tested using a Dunn post hoc test with a Bonferroni adjustment.

Next, to identify the influence of biological parameters on the ingestion of MPs, MFs and APs in *C. sapidus*, a generalized linear model (GLM) with a negative binomial distribution was performed as the Poisson models were over-dispersed. Additionally, the soft tissue (g) was considered as an offset to adjust for the range in soft tissue weights. Here, four parameters were considered: sex (male and female), life stage (adult and juvenile), chela length and condition index (%). The condition index (CI) was calculated following Truchet et al. (2022) as shown in Eq. (1):

$$CI=\frac{Carapace width (mm)}{Total weight (g)} \times 100\%$$

To identify the influence of human impacts on the abundance of ingested items, these models followed the same format as the previous biological GLMs (negative binomial and soft tissue (g) as an offset) and were performed for APs, MPs and MFs. In terms of human impacts, those locations with a protection management strategy were classified as ‘pristine’ while those without any current management requirements were considered non-pristine. Although the location of Addaia in Menorca falls within the Natural Park of Es Grau and is an SPA and SCI site, because it is a harbour area with a coastal population, we did consider this site to be non-pristine. Furthermore, to identify potential sources of MPs and MFs found, the number of drainage pipes and underwater sewage pipelines within a 5-km buffer was calculated for each of the locations (Figure SM1). This was calculated using the available data for the drainage pipes and the georeferenced location of the underwater sewage pipes freely available from Infraestructura de Dades Espacials de les Illes Balears (ideIB, https://ideib.caib.es/visor/). Taking into account this, the final models considered protection status, the number of drainage pipes and the number of sewage pipelines.

### Quality assurance and quality control

Throughout all laboratory steps, 100% white cotton lab coats were worn, minimal personnel was in the laboratory during sampling, and air circulation was minimised to prevent airborne contamination. Before working, all surfaces were cleaned with Milli-Q, and the use of glassware was prioritised. During the chemical digestion process, a KOH blank was included in every sample batch (*n* = 10) to ensure that no MPs or MFs were observed in the liquid reagents. Additionally, all steps in the chemical digestion procedure were covered with aluminium foil. In addition, a glass microfibre filter in an open borosilicate glass Petri dish was placed near the working spaces and routinely checked after each sample for airborne contamination.

## Results

### *C. sapidus* biometrics

A total of 120 individuals were selected from six locations in the Balearic Islands, 20 from each location. The average length for the females was 6.0 ± 0.8, cm and the width was 13.9 ± 2.2 cm, and the males were slightly larger with an average length of 6.5 ± 0.9 cm and a width of 14.3 ± 2.0 cm (Table 2). Additionally, the length of the chela was also longer for males (8.3 ± 1.7 cm) compared to females (6.3 ± 1 cm). By location, the largest individuals were observed in Canyamel in Mallorca and Addaia in Menorca, while the location with the smallest captured individuals was in Torrent de na Borges in Mallorca and Ses Salines in Ibiza (Table 2).

**Table 2:** Summary of the biometrics for the female and male crabs collected at each of the six sampling sites distributed throughout the Balearic Islands. The biometrics include carapace length (cm), carapace width (cm), and chela length (g) for the mean and standard deviation

		Females			Males		
Island	Sampling site	Length (cm)	Width (cm)	Chela (cm)	Length (cm)	Width (cm)	Chela (cm)
Mallorca	S’Albufera	5.6 ± 0.6	12.9 ± 1.9	5.7 ± 0.9	6.2 ± 0.4	13.4 ± 1.0	7.7 ± 1.0
	Canyamel	7.0 ± 0.7	16.6 ± 2.4	7.2 ± 1.1	7.6 ± 0.6	16.6 ± 1.3	10.0 ± 1.0
	Torrent de na Borges	5.1 ± 0.3	12.6 ± 1.3	5.4 ± 0.5	5.7 ± 0.5	12.4 ± 0.9	7.3 ± 1.1
Menorca	Addaia	6.4 ± 0.5	15.0 ± 2.0	7.1 ± 0.7	7.0 ± 0.8	15.4 ± 2.2	9.7 ± 1.4
	Es Grau Menorca	6.0 ± 0.4	13.5 ± 1.4	6.4 ± 0.8	6.9 ± 0.8	15.0 ± 1.6	8.7 ± 2.0
Ibiza	Ses Salines Eivissa	5.5 ± 0.5	13.3 ± 1.6	5.9 ± 0.6	5.8 ± 0.3	12.7 ± 0.7	6.8 ± 0.9
Total		5.9 ± 0.8	13.9 ± 2.2	6.3 ± 1.0	6.5 ± 0.9	14.3 ± 2.0	8.3 ± 1.7

### Microplastic ingestion

#### Classification

**Fig. 2:**
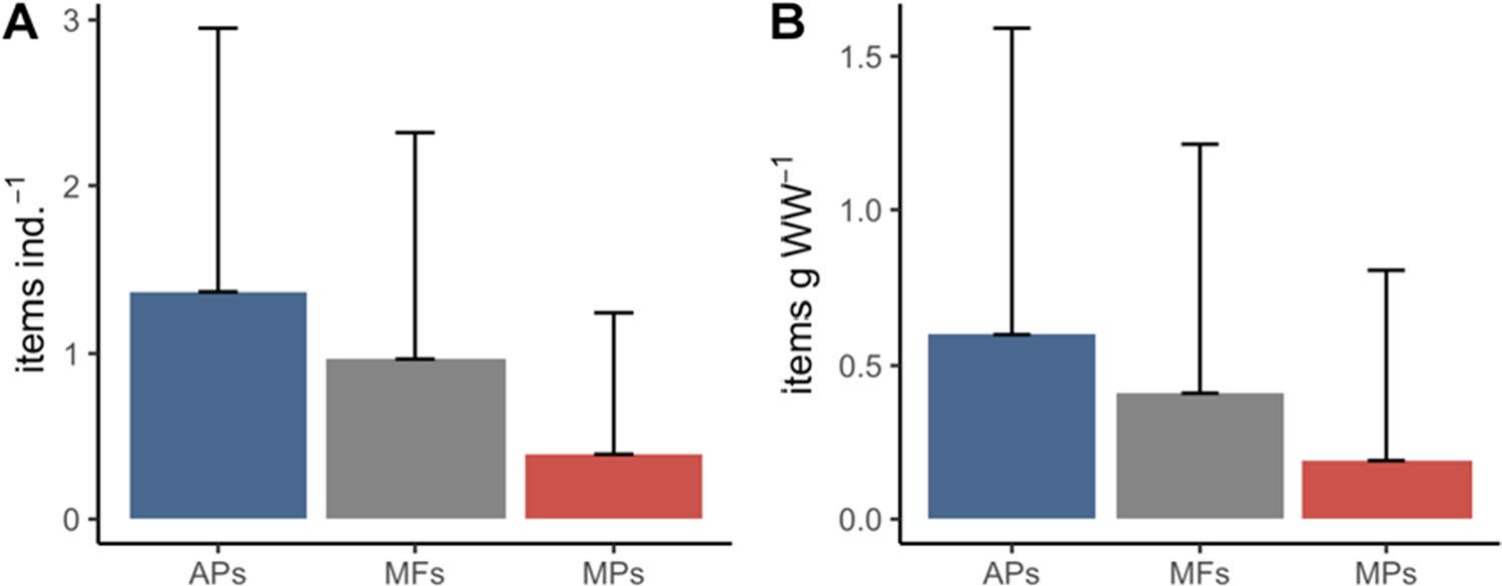
Summary of the average and standard deviation of the number of items ind.^−1^ (**A**) and the number of items g WW^−1^ (**B**) in the digestive cavities of *C. sapidus* in the Balearic Islands for all anthropogenic particles (APs, blue), microfibres (MFs, grey) and microplastic particles (MPs, red). Error bars indicate standard deviation

In terms of particles found in the digestive cavity of *C. sapidus*, 65.8% of the individuals had MP and/or MF particles with an average of 1.4 ± 1.6 APs ind.^−1^, 1.0 ± 1.3 MFs ind.^−1^ and 0.4 ± 0.8 MPs ind.^−1^ (Fig. 2A) and in terms of items per soft tissue, 0.6 ± 1.0 APs g WW^−1^, 0.4 ± 0.8 MFs g WW^−1^ and 0.2 ± 0.6 g MPs WW^−1^. Additionally, significantly more items of MFs were observed compared to MPs for both items ind.^−1^ (MW, *p* < 0.001) and items g WW^−1^ (MW, *p* < 0.001). In Ses Salines, there was one individual with 107 items, with an average number of items to an average of 7.35 ± 23.5 APs ind.^−1^ and 3.1 ± 11.7 APs g WW^−1^ respectively, and as it is an outlier, it was removed from the overall analysis. Considering this, a total of 273 items were recovered and classified by type and colour.

**Fig. 3:**
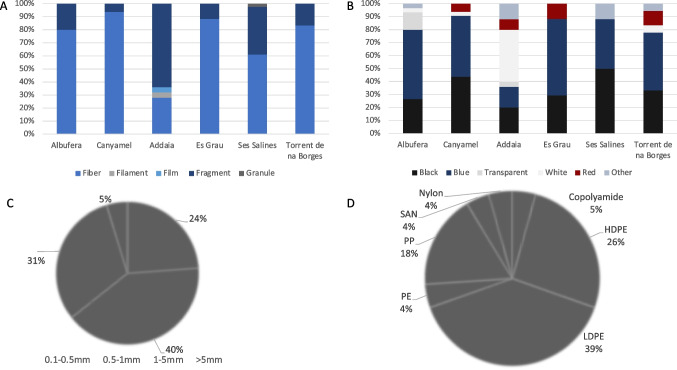
Summary of the physical characteristics of the different items observed by location for: **A** the type of the items (fibre, filaments, film, fragment and granule) and **B** the colour (black, blue, transparent, white, red and other) and **C** size distribution (mm) for all sites

In terms of type, the majority of the items were fibres (71.9%) followed by fragments (26.3%) while filament, film and granules made up less than 2% (Fig. 3A). By location, fibres were the most abundant at all locations with a range between 61 and 93.8% of the items, except Addaia, where only 28% of the items were fibres. The next most common elements were fragments that ranged from 6.3% (Canyamel) to 64% (Addaia). The other less common items, granules (2.4%), were observed in Ses Salines, filaments (4%) in Addaia and films (4%) in Addaia (Fig. 3A). By colour, the most common colour was blue (43.2%) followed by black (35.4%) for all items. The remaining colours (transparent, white, red and other) were just over 20% of the items. There was a slight amount of variability in colours observed by locations. Black and blue were observed at all of the locations (Fig. 3B). Considering the other colours, white was the most abundant in Addaia, while red was observed at all locations except for S’Albufera and Ses Salines. Finally, other colours were observed at all of the locations except for Canyamel and Es Grau (Fig. 3B). In terms of size classification, a total of 42 items were randomly selected for measurements and the most common size of items ranged from between 0.5 and 1 mm (40%) followed by 1 to 5 mm (31%) while the largest fraction (> 5 mm) is the least common (5%) (Fig. 3C). An example of each of the different items identified can be observed in Figure SM2.

A total of 23 items (10%) were characterized by μ-ATR-FTIR. Eight different polymers were detected, of which the most common were polypropylene (PP, 18%), low-density polyethylene (LDPE, 39%) and high-density polyethylene (HDPE, 26%) (Fig. 3D). Additionally, the remaining polymers with fewer frequencies were styrene-acrylonitrile (SAN, 4%), which was a fragment, and copolyimide (5%), polyethylene (PE, 4%) and nylon (4%), which were fibres.

#### Spatial distribution

**Fig. 4:**
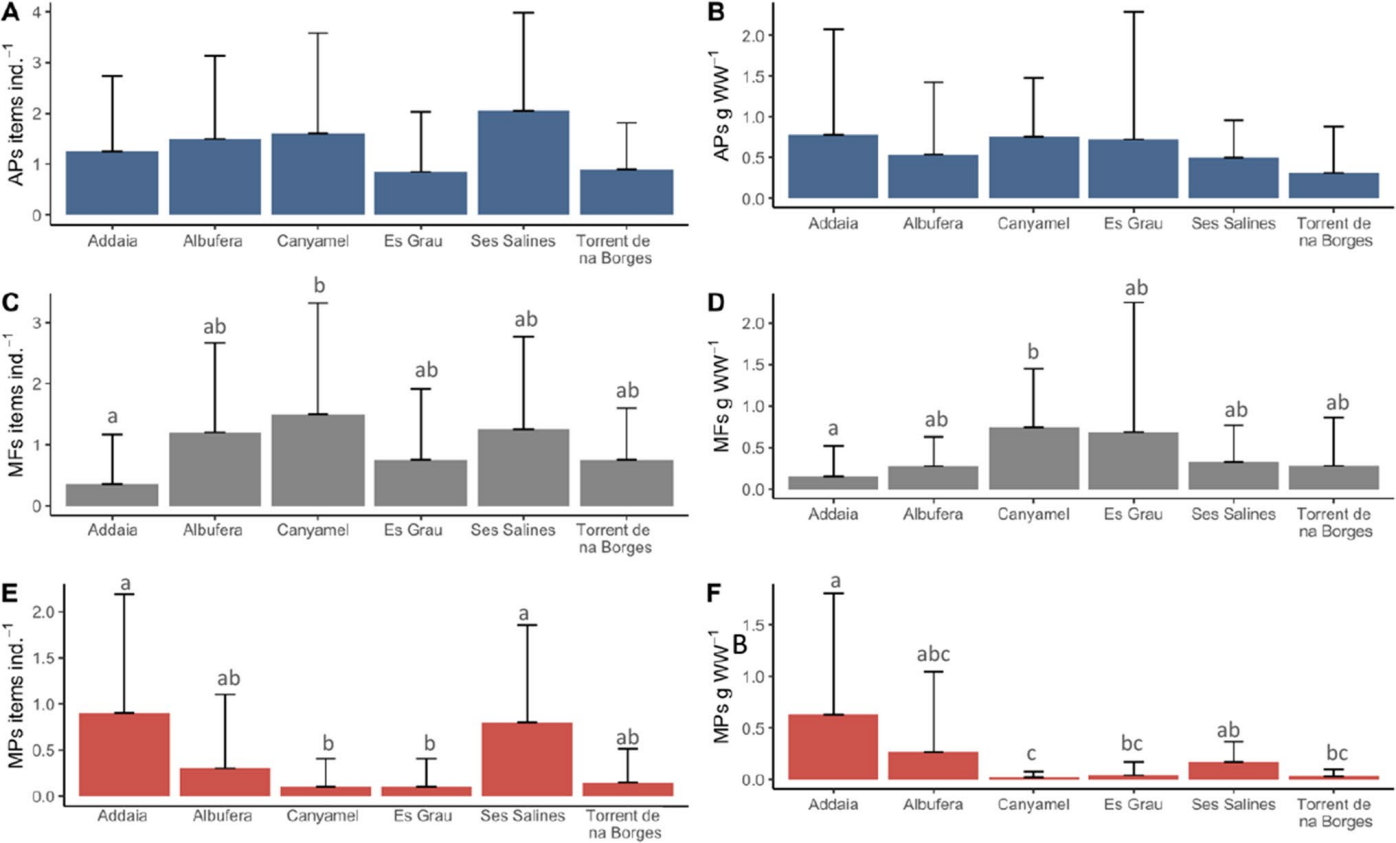
Bar plots of the average and standard deviation of the number of items ind.^−1^ (**A**, **C**, **E**) and the number of items g WW.^−1^ (**B**, **D**, **F**) in the digestive cavities of *C. sapidus* at each location in the Balearic Islands for all anthropogenic particles (APs, blue), microfibres (MFs, grey) and microplastic particles (MPs, red). Different letters indicate significant differences (*p* < 0.05)

In terms of the spatial distribution, no significant differences were observed between locations when considering both MPs and MFs combined (APs, KW, *p* > 0.05). The location with the highest concentration per individual was Ses Salines, with an average of 2.1 ± 1.9 APs ind.^−1^, and the location with the lowest was Es Grau in Menorca with an average of 0.8 ± 1.2 APs ind.^−1^ (Fig. 4A). By number of items g WW^−1^ on the other hand, Canyamel (0.8 ± 0.7 APs g WW^−1^) and Es Grau (0.7 ± 1.5 APs g WW^−1^) had the highest concentrations. When considering MPs and MFs separately, we can observe differences based on location, with significant differences in the number of ingested items ved for MPs (KW, *p* < 0.05) and MFs (KW, *p* < 0.05) (Fig. 4). For MFs, the post hoc analysis revealed significant differences between Addaia in Menorca and Canyamel in Mallorca with an average of 1.5 ± 1.8 MFs ind.^−1^ and 0.2 ± 0.4 MFs g WW^−1^ in Addaia and an average of 0.4 ± 1.9 MFs ind.^−1^ and 0.7 ± 0.7 MFs g WW^−1^ in Canyamel (Fig. 4 C and D). Regarding differences between locations for the ingestion of MPs, Canyamel (0.10 ± 0.3 MPs ind.^−1^ and 0.02 ± 0.05 MPs g WW^−1^) had the lowest concentrations and Addaia (0.9 ± 1.2 MPs ind.^−1^ and 0.6 ± 1.2 MPs g WW^−1^) having the highest number of items ingested. A spatial representation of the connectivity from the post hoc results can be observed in Figure SM3.

### Biological factors

**Fig. 5:**
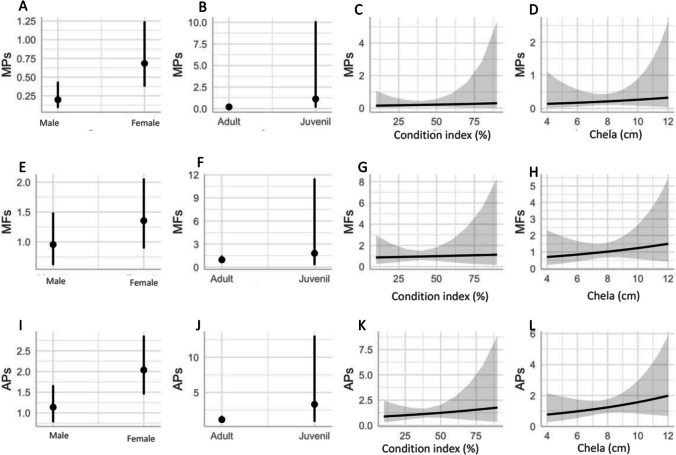
Results from the generalised linear model’s marginal effects of predictors that take into account the biological parameters of each model’s ingestion take into account sex (male and female), life stage (adult and juvenile), condition index per cent and chela length (cm) for microplastics (MPs) **A**–**D**, microfibers (MF) **E**–**H** and all particles (APs) **I**–**L**

To determine potential biological factors (sex, life stage, condition index and chela length) that can influence ingestion, three GLMs were performed for each of the particle types (MPs, MFs and APs). For MP and AP models, females ingested significantly more items than males (Fig. 5E, SM4 and Table SM1). Although it was not statistically significant, the juveniles did slightly consume more items than the adults in terms of stage of life (Fig. 5J), and only a weak positive relationship was observed that was not determined to be significant with the condition index (Fig. 5K). The length of the chela was also found to have a positive relationship, but this was also determined to be insignificant (Fig. 5L).

### Human impacts

**Fig. 6:**
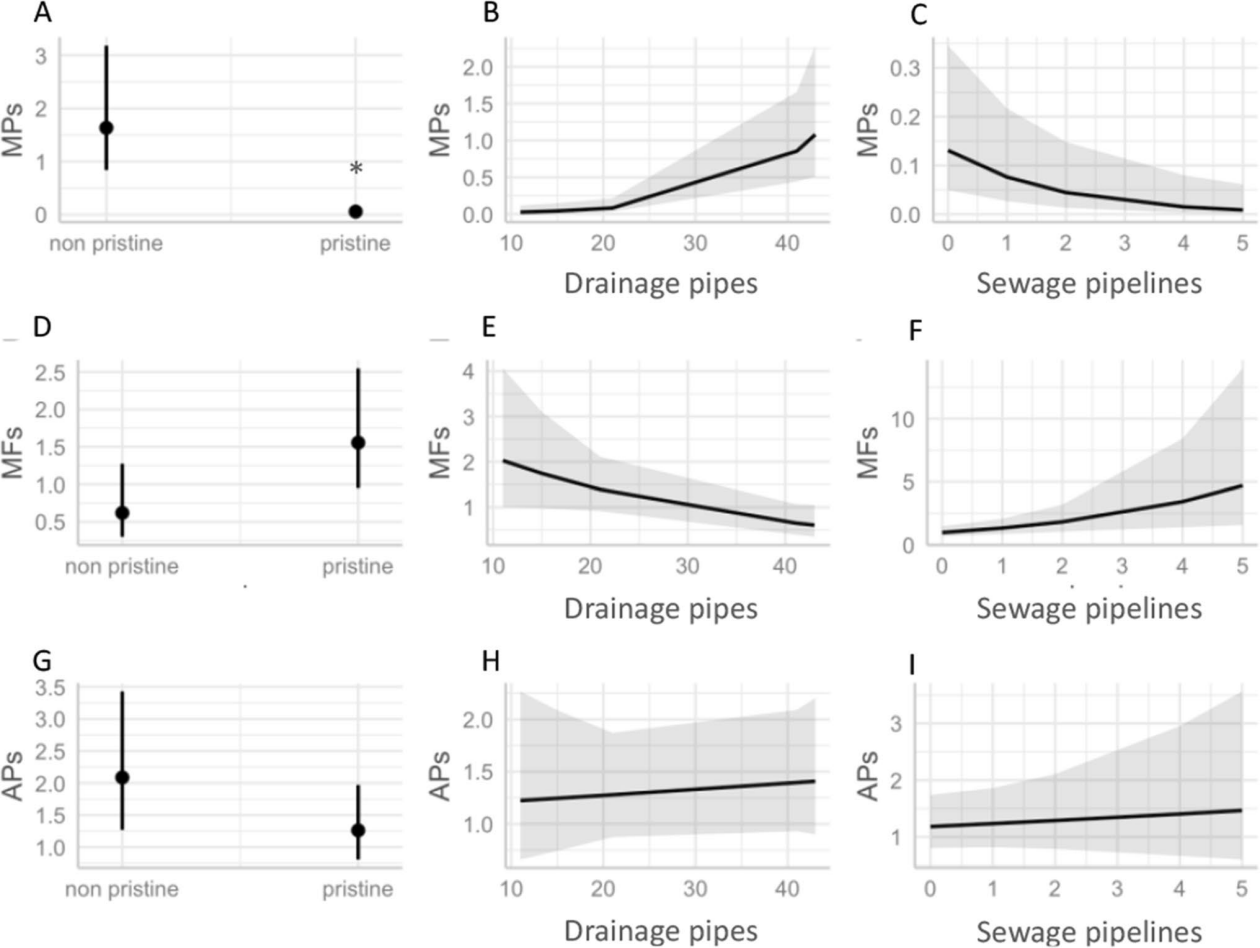
Results from the generalized linear model’s marginal effects taking into account protection status (non-pristine and pristine), drainage pipes and sewage pipelines for microplastics (MPs) **A**–**C**, microfibers (MF) **D**–**F** and all particles (APs) **G**–**I**

To determine possible human impacts that can contribute to the ingestion of MFs and MPs in *C. sapidus*, the protection status of the area was analysed in addition to calculating the number of drainage pipes and sewage pipelines within 5 km of each of the survey sites. Results from the GLMs were model-dependent. For example, for the MP model, significantly more items were ingested in non-pristine areas than in pristine areas (Fig. 6A–C; Table SM2). Furthermore, *C. sapidus* were more likely to ingest MPs within proximity of a higher number of drainage pipes while the opposite was observed in the MF model, where a positive relationship was observed with an increased number of sewage pipelines (Fig. 6D–F and Table SM2). For the final model considering APs (combination of MPs and MFs), no significant differences were observed between pristine and non-pristine areas; this is an indication that when considering both types of particles, there is a homogeneous distribution throughout the region regardless of protection status (Fig. 6G–I; Table SM2). An increase in the number of both drainage pipes and sewage pipelines was shown to have a strong positive connection, suggesting that both may be related to an increase in the abundance of items found in the *C. sapidus* stomach contents.

## Discussion

Growing research highlights the presence of MPs and MFs in estuaries, wetlands and lagoons globally. In the present study, we highlight the presence of these items in the blue crab *C. sapidus*, which has established itself in nearshore and coastal habitats including salt marshes and estuaries throughout the archipelago of the Balearic Islands in the western Mediterranean Sea. Here, we identify the frequency of occurrence of MPs in stomach contents as an indicator of contamination in these areas.

### Microplastic occurrence

To date, only a few studies have reported the ingestion of MPs and MFs in *C. sapidus.* The results of this study reported concentrations similar to those observed by Waddell et al. (2020) where MPs were observed in 35.9% of the individuals sampled, with an average of 0.87 items per crab. These concentrations are lower than those observed in other studies, such as the first report of MP ingestion in *C. sapidus* from the Mediterranean, where an average of 11.0 11.0 ± 1.85 MPs⋅g^−1^ was observed in a natural lagoon in Albania, in the Adriatic Sea (Aliko et al. 2022). In the Gulf of Mexico, Capparelli et al. (2022) reported the presence of 37.9 MPs g^−1^ in the gills of *C. sapidus*, while only 8.62 MPs g^−1^ were observed in the digestive tract, of which the majority of the items were fibres. For other crab species, in the present study, slightly higher concentrations were found when compared to the tidal crabs *Neohelice granulat*a (0.36 ± 0.25 g WW^−1^), *Cyrtograpsus angulatus* (0.19 ± 0.11 g WW^−1^) and *Leptuca uruguayensis* (0.06 ± 0.07 g WW^−1^) (Table 3).

**Table 3:** Summary comparison of previous studies identifying the ingestion of microplastics and microfibres in different species of crabs. NA indicates the information was not available and ND indicates the analysis was not determined

Study	Location	Species	Items individual^−1^	Items g^−1^ (wet weight)	Method used	Tissue analysed
This study	Balearic Islands, Spain	*Callinectes sapidus*	2.1 ± 1.5	0.4 ± 1.04	10% KOH	Stomach
Aliko et al. (2022)	Albania	*Callinectes sapidus*	43 to 50	10.75 ± 1.4 to 12.5 ± 2.3 (range)	10% KOH	Stomach
Capparelli et al. (2022)	Gulf of Mexico	*Callinectes sapidus*		37.9 (mean)	30% H_2_O_2_	Gills
				8.62 (mean)	30% H_2_O_2_	Digestive Tract
Waddell et al. (2020)	Corpus Christi Bay, Gulf Coast	*Callinectes sapidus*	0.44 to 0.72	NA	30% H_2_O_2_	Stomach
Horn et al. (2019)	California Coast	*Emerita analoga*	0.65 ± 1.64	NA	Visual separation	Digestive Tract
Truchet et al. (2022)	Buenos Aires, Argentina	*Neohelice granulata*	NA	0.36 ± 0.25	10% KOH	Gut
			NA	1.5 ± 1.7	10% KOH	Carapace
			ND	ND	10% KOH	Eggs
			NA	1 ± 1	10% KOH	Gills
		*Cyrtograpsus angulatus*	NA	0.19 ± 0.11	10% KOH	Gut
			NA	0.67 ± 0.52	10% KOH	Carapace
			ND	ND	10% KOH	Eggs
			NA	0.19 ± 0.11	10% KOH	Gut
			NA	0.11 ± 0.07	10% KOH	Gills
		*Leptuca uruguayensis*	NA	0.06 ± 0.07	10% KOH	Gut
			NA	0.11 ± 0.07	10% KOH	Carapace
			NA	4 ± 2	10% KOH	Eggs
			NA	0.11 ± 0.07	10% KOH	Carapace
			NA	0.17 ± 0.14	10% KOH	Gills

Fibres and fragments are the two primary MP types found in the invasive *C. sapidus*. The predominance of fibres and fragments is in agreement with Aliko et al. (2022), where the type of items in the stomach contents was more similar to the concentrations of water than the sediment, suggesting that *C. sapidus* is selectively ingesting the MPs based on their shape or that they are more readily available in the water column. In terms of size distribution, the most common size class was items between 0.5 and 1 mm which is a slightly larger size class compared to Aliko et al. (2022) and other studies where the predominant size class in *C. sapidus* and *Carcinus aestuarii* was 0.1–0.5 mm.

Regarding polymer type, the most common polymers observed were LDPE and HDPE. This is in agreement with a previous study on polymer characterization of *C. sapidus* by Aliko et al. (2022) where the most common items were also HDPE, LDPE in addition to PE and PP. Typically, PP, HDPE and LDPE are more commonly found in the water column and on the sea surface (Compa et al. 2020; Rios-Fuster et al. 2022) while PET has a higher density and is more commonly found on the seafloor. The findings demonstrate the wide range of plastic types that can be found in the environment and considering *C. sapidus* is a benthic forager and opportunistic feeder, the results highlight its exposure to particles that can be found not only on the seafloor but also in the water column and on the water’s surfaces. Considering the variety of types of polymers observed in *C. sapidus*, this may be indicative of a possible presence of polymers in estuaries and coastal areas of the Balearic Islands.

### Spatial distribution

Ingestion of APs at various locations in the Balearic Islands provides evidence both for the presence of APs in the immediate environment and their varying bioavailability. Previous environmental studies (Compa et al. 2020; Ruiz-Orejón et al. 2019) have emphasised the prevalence of MP floating on the sea surface of the Balearic Islands while MFs are increasingly more evident in seafloor sediments and the water column (Fagiano et al. 2023). More research is needed on MFs in the Balearic Islands, but many fibres were found in the sea surface waters of Cabrera National Park, and they were also one of the main types of items found in the stomach contents of several bioindicator species studied in the same region (Compa et al. 2022; Fagiano et al. 2022a). As a result, taking into account the homogeneous distribution of AP items also provides a hint as to how common the items are across various systems. This has also been observed in samples collected on the sea surface in the Adriatic (Arcangeli et al. 2018) in addition to plastic items found on the seabed along the coasts of Slovenia and Italy (Strafella et al. 2015). Considering this, benthic species can be considered key indicators for plastic particles in coastal areas.

### Biological traits

In this study, although *C. sapidus* females ingested a slightly higher abundance of MP compared to males, it was only found to be weakly significant. In a previous experimental study on the European hermit crab (*Pagurus bernhardus*), behavioural differences were observed in females after exposure to MPs, which can make them more vulnerable if threatened by a predator (Mcdaid et al. 2023). In a field study by Stasolla et al. (2016) on the invasive crab *Charybdis longicollis*, and three crab species representing different habitats in Truchet et al. (2022), no significant differences were observed between males and females which are in agreement with this study. Considering the few studies on *C. sapidus*, future studies would benefit from incorporating differences considering sex.

The presence of MPs and MFs in the gastrointestinal tracts of crabs may be determined by their ecological characteristics. For example, Capparelli et al. (2022) found a higher occurrence of MPs in the sedentary and carnivorous *Menippe mercenaria* compared to *C. sapidus*, which is identified more as a free swimmer with an omnivorous diet. In a similar study with different species of intertidal crabs, Truchet et al. (2022) observed that overall, the omnivorous burrower *Neohelice granulata* showed the highest prevalence of MPs and MFs (gills, gut, carapace and eggs), although in the gut, the deposit feeder *Leptuca uruguayensis* has the highest occurrence in their stomach contents with an average of 0.36 ± 0.25 MPs⋅g^−1^ WW. These variations could be due to the greater propensity for filter feeding of *L. uruguayensis*, which has been found to consume more APs than other species with a wider variety of eating habits.

In terms of *C. sapidus* feeding ecology, Belgrad and Griffen (2016) determined in a laboratory experiment that crabs whose diets consisted entirely of animal tissue had lower mortality and consumed significantly more food than crabs whose diets consisted entirely of seaweed; additionally, seaweed diets produced a decrease in hepatopancreas lipid content and an increase in crab aggression when compared to a carnivorous animal diet. Considering this, more research into the interactions between the diets of *C. sapidus* and microplastics is needed.

### Human impacts

Regarding human impacts, MP abundances were found to be higher in non-pristine places, MF abundances were found to be higher in pristine areas, and AP abundances were found to be similar in both areas. In terms of drainage pipes and sewage pipelines, similar results are observed. The GLM results indicated that higher ingestion of MPs was associated with an increase in the number of drainage pipes which is also in agreement with Bigalke et al. (2022) where high concentrations of MPs were linked to drainage pipes near agricultural areas. The drain pipes mostly contain storm-water runoff that has been diverted from the streets, gutters, channels, etc. because they do not undergo any sort of treatment, making it more likely that fragmented MPs will reach coastal areas from these outlets. A recent study by Treilles et al. (2021) highlighted that a median of 29 MPs items/L was observed compared to a median of 1.9 MFs items/L in an urban environment. On the other hand, in terms of MFs, higher abundances were observed in pristine areas associated with a higher number of sewage pipelines. In terms of sewer pipelines, they go through a wastewater treatment plant where the majority of pollutants are removed. Considering the size and form of MFs, they are difficult to remove during the wastewater treatment process and often end up being released with the treated water. In this study, results from the GLM indicated that higher ingestion of MFs was associated with an increased number of sewage pipelines. A recent study by Herzke et al. (2021) highlighted that small settlements can introduce billions of MF particles annually. Furthermore, when considering both MPs and MFs, there was a positive relationship between an increased number of drainage pipes and sewage pipelines, an indication that the presence of both types of point source pollutants is potential source of both types of items. Given that the Balearic Islands are a popular tourist destination and that most visitors stay in coastal areas, sewage and drainage outlets need to be monitored for the release of these particles, especially in the summer.

### Future research

Despite growing interest in the implications of MPs and MFs in the marine environment, there are currently few studies that focus on crab species. In the following section, we address two important research gaps. One of them is the potential human health impacts in fisheries resource management, and the second is its potential as a contributor to these items in the marine environment. The potential for eradication of invasive crabs from the marine environment is highlighted by Mancinelli et al. (2017) as being difficult due to the need for detailed information on the occurrence and abundance of populations in addition to connectivity. Since *C. sapidus* is a valuable resource for fisheries in many regions of the world, including Spain, where it is listed as a commercial fish species (BOE-A-2016–3357) and has not been labelled an invasive alien species, there is increasing interest in capturing it in the Mediterranean Sea on a regional level. Thus, *C. sapidus* species could be proposed as an indicator species for MP pollution in other regions considering its high adaptability and ability to thrive in different habitats. Additionally, accurate assessments of the ecological and economic effects on ecosystem services are still required, both as a barrier to other traditional fisheries and as a benefit as a new shellfish product, particularly in the case of *C. sapidus*. Future studies need to integrate potential effects on human impacts, especially considering the elevated number of individuals in this study and previous studies where the ingestion of MPs and MFs items was observed.

Another factor is how invasive crab species interact with plastic and other anthropogenic debris in the marine environment. Until now, studies primarily focus on MPs as a vector for introducing species in the taxa Arthropoda, Annelida and Mollusca and dispersing them to new environments (García-Gómez et al. 2021). An estimated 5% of invasive species have been identified as entering new environments from colonizing MPs, and even fewer studies have documented the interaction of MPs with invasive species (García-Gómez et al. 2021). Several studies have measured the effects that MP ingestion can have on invasive species. For example, Kalinkina et al. (2022) observed a preferential particle size of 100 ± 5 µm in the freshwater amphipod *Gmelinoides fasciatus* and particles in sediment compared to those in suspension. Crabs in general have a unique feeding strategy, as they use the chelipeds as a tool to tear food and then eat it and if they lose their chelipeds, they will use their oral appendages to break up food particles (Oliveira et al. 2015). Although the length of their chelas was not a contributing factor in the number of items ingested in this study and considering that these items were observed in the gastrointestinal tracts of *C. sapidus*, they can become a contributor to the fractioning and degradation of MP and MF in the marine environment. In a controlled laboratory experiment, sea urchins, a related bioeroder species, were found to produce up to 91.7 plastic fragments on average over 10 days (Porter et al. 2023). Considering this, the increasing presence of *C. sapidus* in the Balearic Islands may contribute increase in the abundance of plastic particles, especially within the size range of 0.5–1 mm.

## Conclusions

This study reports the widespread presence of MP and MF particles even in pristine areas that are currently managed under various protection statuses by highlighting their prevalence in the stomach contents of the invasive *C. sapidus* throughout the coastal community of the Balearic Islands. Additionally, it is critical to manage not only invasive species but also incorporate methods to check for APs in these regions because MPs and MFs are a pollutant present at many of the survey locations which are covered by management conservation plans. By including both factors in management plans, it is possible to minimize their negative impacts on the environment, promote the health and sustainability of ecosystems and provide targeted and coordinated approaches to mitigating their effects. Taking into account the increase in observations of *C. sapidus* in coastal communities throughout the Mediterranean Sea, these results serve as a baseline for future studies.

### Supplementary Information

Below is the link to the electronic supplementary material.

Supplementary file1 (DOCX 26917 KB)

## Data Availability

Data will be made available upon reasonable request.
