# Supplementary material for: Ingestion of microplastics and microfibers by the invasive blue crab Callinectes sapidus (Rathbun 1896) in the Balearic Islands, Spain
Source: Environ Sci Pollut Res Int. 2023 Nov 4;30(56):119329–42. doi: 10.1007/s11356-023-30333-x (PMC10698140; doi:10.1007/s11356-023-30333-x)
Supplement: Supplementary file 1 — Supplementary file1 (DOCX 26917 KB) [file 11356_2023_30333_MOESM1_ESM.docx]

**Supplementary Material**

Figure SM1 Maps of the number of drain pipes and sewage pipelines in the Balearic Islands for Mallorca (A), Ibiza (B) and Menorca (C). The points indicate drain pipes and the brown lines indicate sewage pipelines. The 5 km buffer from each sampling site has been represented with a red circle.

Figure SM2 Representative images of the items identified according to different types: A) fibre, B) fragment, C) filament and D) pellet.


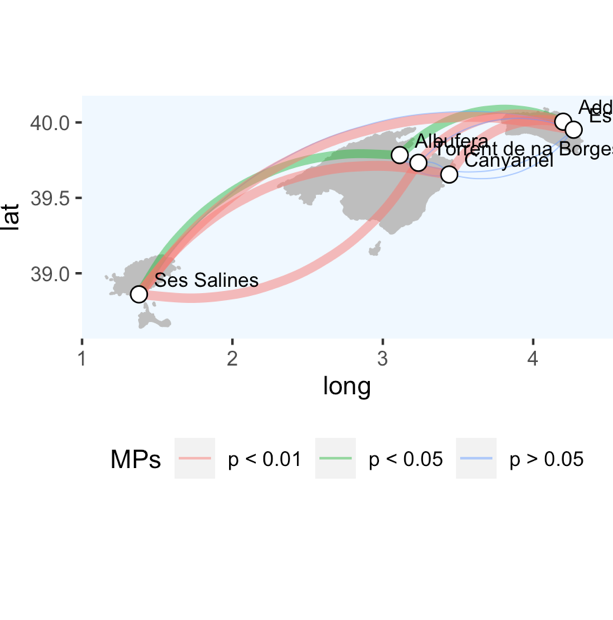

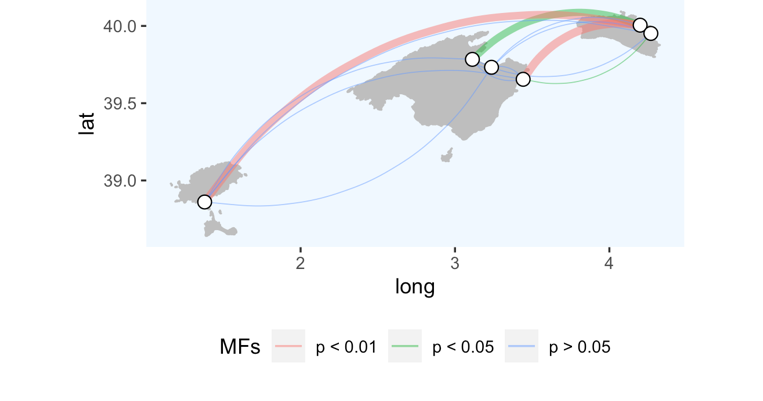


Figure SM3 Maps of the results from the pairwise comparison of the post-hoc tests indicated the significant differences in MPs and MFs between sites for *C. sapidus* at each location in the Balearic Islands. No significant differences were observed between APs and therefore only maps for MPs (A) and MFs (B) are provided. Red indicates p < 0.01, green indicates p < 0.05 and blue indicates p > 0.01. The weight of the line is the p-value given from the post-hoc test.


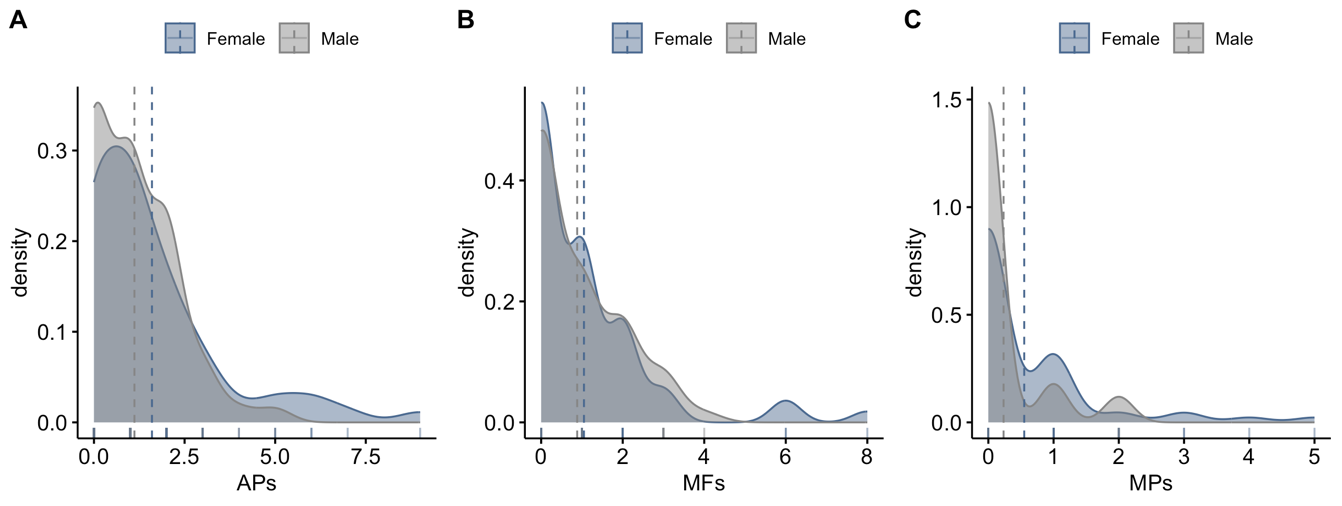


Figure SM4 Summary of the density of ingested items between female (blue) and male (grey) *C. sapidus* for APs (A), MFs (B) and MPs (C). The dashed lines indicate the mean abundance of items.

Table SM1 Summary of the results from the generalized linear models for microplastics, microfibers and all particles taking into consideration sex, life stage, chela and the soft tissue as an offset. Statistical analysis GLM: *p > 0.05, ** p > 0.01, *** p > 0.001.

Table SM2 Summary of the results from the generalized linear models taking into consideration human impacts for microplastics, microfibers and all particles taking into consideration protection status (pristine/non pristine), drainage pipes, sewage pipelines and the soft tissue as an offset. Statistical analysis GLM: *p > 0.05, ** p > 0.01, *** p > 0.001.
